# A rare precursor of gastric tumor

**DOI:** 10.1093/gastro/gou029

**Published:** 2014-06-13

**Authors:** Khalid A. Alkimawi, Asim Shuja

**Affiliations:** ^1^Department of Gastroenterology, Tufts Medical Center, Boston, MA 02111, USA and ^2^Department of Medicine, St. Elizabeth’s Medical Center, Brighton, MA 02135, USA

**Keywords:** gastritis, gastric tumor, gastritis cystical profunda

## Abstract

Gastritis cystica profunda (GCP) is a rare tumor precursor which occurs more commonly in patients who have undergone previous gastric surgery. The non-specific symptoms and radiographic appearance of this tumor mimic those of other hyperproliferative conditions, making diagnosis difficult. This is a pre-malignant condition and may lead on to carcinoma of the stomach. Here we report a 57-year-old female with no previous gastric surgeries, who presented to us with epigastric abdominal pain. Her work-up included an upper endoscopy, which revealed fundic polyps. Her fundal biopsies were consistent with GCP.

## INTRODUCTION

Gastritis cystica profunda (GCP) is a rare tumor precursor, which occurs more commonly in patients who have previously undergone gastric surgery. The histological findings—of cystic glandular inclusions with a connective tissue laden submucosa—were first described by Littler and Gleibermann in 1972 [1]; however it was not until 1981 that Franzin and Novelli coined the term *gastritis cystica profunda*, and described fifteen cases of GCP that were initially confused with other gastric pathologies [2]. Since that time, very few additional cases of GCP have been reported and, in these instances, GCP was initially thought to represent Ménétrier’s disease [3–5], gastric adenocarcinoma [6, 7], inverted hyperplastic polyps [8], and other pathologies. This report describes a case of GCP in the fundus of a surgery-naïve 57-year-old female who presented with epigastric pain.

## CASE PRESENTATION

**Figure 1.**
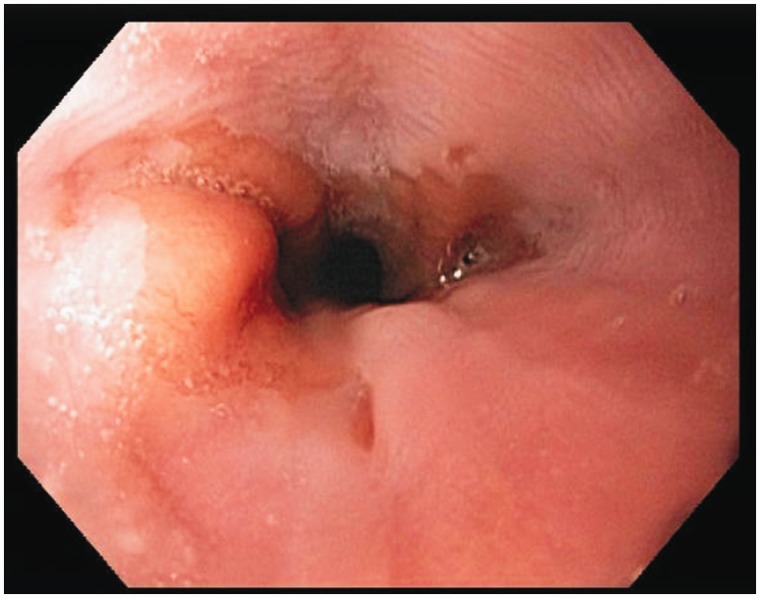
Irregular gastro-esophageal junction, biopsies were negative for Barrett's Esophagus.

**Figure 2.**
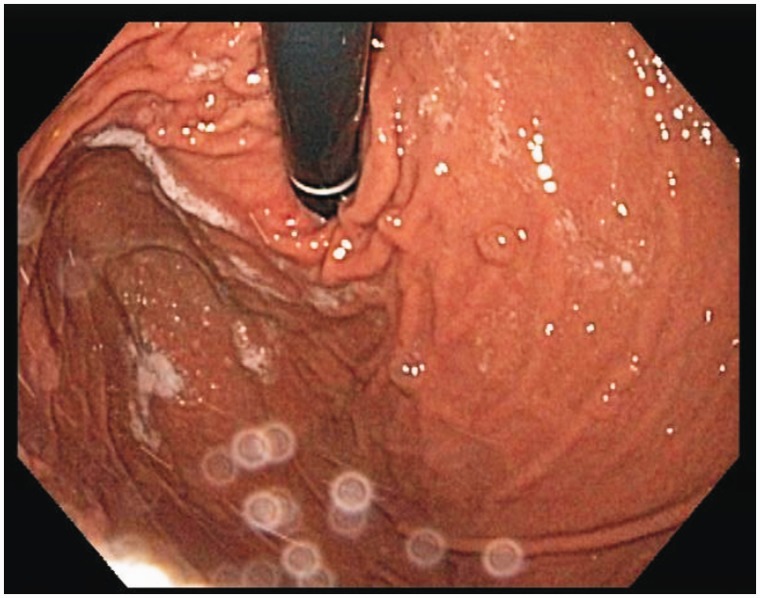
Multiple small polyps were found in the fundus of stomach.

**Figure 3.**
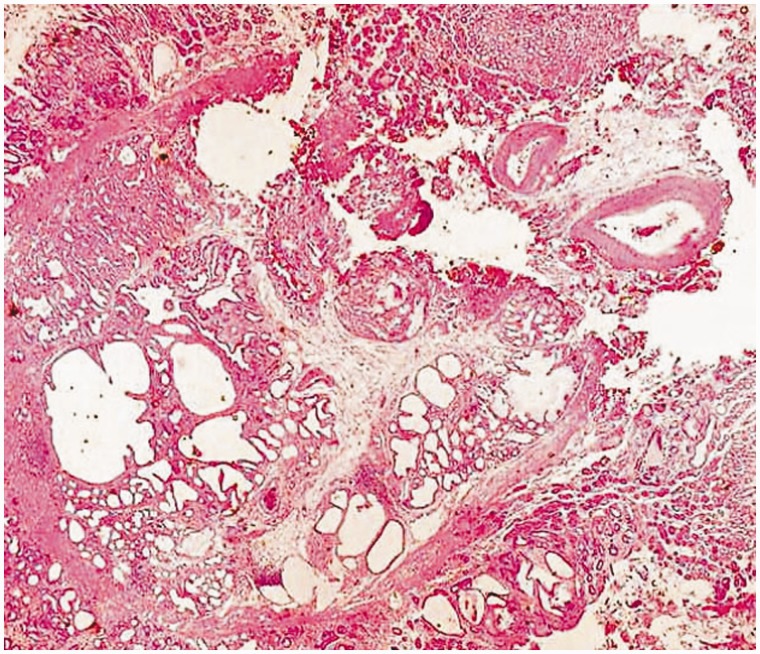
Cold forceps biopsies showed cystic down-growth of gastric glands into the submucosa of the stomach.

Our subject was a 57-year-old Caucasian female, who initially presented to us in 2011 with progressive heartburn. Her diet and behavior were reviewed; most triggers were eliminated and she was started on a proton pump inhibitor once a day. The frequency was changed to twice a day and all heartburn symptoms resolved by early 2012. She had no previous medical or surgical history, did not drink alcohol or consume tobacco and had no family history of cancer. She had had a right upper quadrant ultrasound many years ago for similar abdominal pain and was found to have a gall bladder polyp. She presented to us again with abdominal pain early in 2013. We repeated her ultrasound, which showed a stable gall bladder polyp, with no increase in size. Her blood work, including a complete blood count, basic metabolic profile and liver function tests, were all normal. Cardiac evaluation excluded ischemic heart disease. On this presentation, she had a faint epigastric pain. The abdominal examination did not elicit any tenderness, but she did report early satiety and weight loss; we therefore performed an upper endoscopy and colonoscopy, which was normal, apart from a few diverticula. The upper endoscopy showed an irregular gastro-esophageal junction (Figure 1); biopsies did not show Barrett's Esophagus. On retroflexion, a few fundic polyps were noted but no mass lesions were observed (Figure 2). Random biopsies were obtained; the pathological findings were consistent with GCP (Figure 3). At 3-month follow-up, she was not complaining of any epigastric pain and had improvement in and had gained weight. We will repeat her endoscopy annually for screening purposes.

## DISCUSSION

GCP is a condition characterized by benign, cystic down-growth of gastric glands into the sub-mucosa of the stomach [9]; this is a well-described but rare phenomenon following gastric surgery [10]. The pathogenesis of GCP is probably due to chronic ischemia and inflammation occurring at a previous gastric surgery suture site [2]. Disruption of the integrity of the muscularis mucosa causes the migration of epithelial contents into the sub-mucosa, with consequent atrophic gastritis, intestinal metaplasia and cystic dilatation of the gastric glands [2]. GCP has been described in a patient with a history of gastric ulcer, who was treated with H_2_ blockers [11]. This indicates that GCP can occur after exposure of the gastric mucosa to an insult. Examples could include chronic mucosal inflammation, which subsequently leads to hyperplastic and metaplastic changes, and increased risk of progression towards carcinoma. A defined treatment strategy for GCP has not been well described, given the rarity of the lesions and the difficulty in diagnosing them. GCP could generally resemble the benign fundic gland polyps as in this patient, but the histology is different.

Morphologically, fundic gland polyps are different from GCP, as the former are usually numerous. Histologically, fundic polyps show characteristic dilatations of oxyntic glands, with only minimal stroma, and the surface foveolar epithelium is either normal or focally flattened. Benign fundic gland polyps usually have both superficial, as well as deep, dilations. They usually have no association with prior gastric surgery [12].

Fundic gland polyps can be sporadic or found in association with other conditions, such as familial adenomatous polyposis (FAP), attenuated FAP, etc. In sporadic cases of fundic gland polyps, more than 90% of patients have activating mutations in the β-catenin gene, which is not the case in pathogenesis of GCP [13]. In FAP, the abnormality is a mutation in the APC gene, resulting in its deactivation. Attenuated FAP can occur from other mutations in the APC gene, and causes a phenotype wherein colonic polyps may be few in number [14]. As far as the clinical presentation of GCP and fundic gland polyps is concerned, there is no major difference and findings are mostly subtle, such as mild abdominal pain, nausea or other symptoms if associated with other conditions like FAP [15, 16].

Although there is no definite evidence that GCP may be pre-cancerous, dysplastic changes in submucosal glands in GCP suggest that it may have a malignant potential. GCP may represent a paracancerous lesion, and there have been some reports of GCP associated with cancer. According to Iwanaga *et al.*, GCP accompanies 3% of gastric carcinoma and several cases showing its relationship with cancer have been reported. There was also a study of histological features, stating that GCP had some characteristics of malignancy, such as metastatic and dysplastic alteration. Expression of Ki-67, p53, and p21 in GCP were as high as in cancer tissue [16].

## CONCLUSIONS

We present a case of GCP presenting as epigastric pain in a patient who had never undergone gastric surgery. GCP may be a pre-cancerous lesion, or can be associated with a cancer; for this reason a repeat endoscopy is therefore warranted. The pathogenesis of GCP is related to ischemia, chronic inflammation and the presence of a foreign body at the previous surgical site. GCP patients have not been followed-up for long periods of time; thus screening, treatment and timings of excisions are unknown. GCP may resemble benign fundic gland polyps but differ histologically. More research needs to be conducted in the pathogenesis of GCP as they occur even in individuals who have not undergone surgery.

**Conflict of interest:** none declared.

## References

[1] Littler ER, Gleibermann E (1972). Gastritis cystica poly-posa. (Gastric mucosal prolapse at gastroenterostomy site, with cystic and infiltrative epithelial hyperplasia). Cancer.

[2] Franzin G, Novelli P (1981). Gastritis cystica profunda. Histopathology.

[3] Béchade D, Desramé J, Algayres JP (2007). Gastritis cystica profunda in a patient with no history of gastric surgery. Endoscopy.

[4] Lim JK, Jang YJ, Jung MK (2010). Ménétrier disease manifested by polyposis in the gastric antrum and co-existing with gastritis cystica profunda. Gastrointest Endosc.

[5] Okada M, Iizuka Y, Oh K (1994). Gastritis cystica profunda presenting as giant gastric mucosal folds: the role of endoscopic ultrasonography and mucosectomy in the diagnostic work-up. Gastrointest Endosc.

[6] Moon SK, Kim KO, Park SH (2010). Gastritis cystica profunda accompanied by multiple early gastric cancers. Kor J Gastroenterol.

[7] Tsuji T, Iwahashi M, Nakamori M (2008). Multiple early gastric cancer with gastritis cystica profunda showing various histological types. Hepatogastroenterology.

[8] Yamashita M, Hirokawa M, Nakasono M (2002). Gastric inverted hyperplastic polyp. Report of four cases and relation to gastritis cystica profunda. APMIS.

[9] Kurland J, DuBois S, Behling C (2006). Severe upper-GI bleed caused by gastritis cystica profunda. Gastrointest Endosc.

[10] Qizilbash AH (1975). Gastritis cystica and carcinoma arising in old gastrojejunostomy stoma. Can Med Assoc J.

[11] Itte V, Mallick IH, Moore PJ (2008). Massive gastrointestinal haemorrhage due to gastritis cystica profunda. Cases J.

[12] Shaib YH, Rugge M, Graham DY (2013). Management of gastric polyps: an endoscopy-based approach. Clin Gastroenterol Hepatol.

[13] Abraham SC, Nobukawa B, Giardiello FM (2001). Sporadic fundic gland polyps: common gastric polyps arising through activating mutations in the β-catenin gene. Am J Pathol.

[14] Burt RW (2003). Gastric fundic gland polyps. Gastroenterology.

[15] Church JM, McGannon E, Hull-Boiner S (1992). Gastroduodenal polyps in patients with familial adenomatous polyposis. Dis Colon Rectum.

[16] Moon SY, Kim KO, Park SH (2010). Gastritis cystica profunda accompanied by multiple early gastric cancers. Kor J Gastroenterol.

